# What interventions should we implement in England's mental health services? The mental health implementation network (MHIN) mixed-methods approach to rapid prioritisation

**DOI:** 10.3389/frhs.2023.1204207

**Published:** 2023-08-11

**Authors:** Shalini Ahuja, Lawrence Phillips, Caroline Smartt, Sundus Khalid, Tina Coldham, Laura Fischer, Sarah Rae, Nick Sevdalis, Annette Boaz, Sarah Robinson, Fiona Gaughran, Zoe Lelliott, Peter Jones, Graham Thornicroft, Jayati-Das Munshi, Colin Drummond, Jesus Perez, Peter Littlejohns

**Affiliations:** ^1^ Methodologies Research Division, Florence Nightingale Faculty of Nursing Midwifery and Palliative Care, King's College London, London, United Kingdom; ^2^ Department of Management, London School of Economics and Political Science, London, United Kingdom; ^3^ Department of Health Service & Population Research, School of Mental Health & Psychological Sciences, King's College London, London, United Kingdom; ^4^ Participation Involvement & Engagement Advisor at NIHR (National Institute for Health Research), London, United Kingdom; ^5^ Independent Expert by Experience, and Patient Community Involvement and Engagement Participation (PCIEP) Lead, co-Lead Workstream 2 (Patient and Public Involvement), London, United Kingdom; ^6^ Department of Psychological Medicine, National University of Singapore, Singapore, Singapore; ^7^ Department of Health Services Research and Policy, London School of Hygiene and Tropical Medicine, University of London, London, United Kingdom; ^8^ Eastern Academic Health Science Network, Cambridge, United Kingdom; ^9^ Social and Psychiatric Epidemiology, Institute of Psychiatry, Psychology and Neuroscience, King's College London, London, United Kingdom; ^10^ Department of Psychiatry, University of Cambridge, Cambridge, United Kingdom

**Keywords:** mental health priorities, mental health implementation, evidence based mental health, England, decision making

## Abstract

**Introduction:**

Setting mental health priorities helps researchers, policy makers, and service funders improve mental health services. In the context of a national mental health implementation programme in England, this study aims to identify implementable evidence-based interventions in key priority areas to improve mental health service delivery.

**Methods:**

A mixed-methods research design was used for a three step prioritisation approach involving systematic scoping reviews (additional manuscript under development), expert consultations and data triangulation. Groups with diverse expertise, including experts by experience, worked together to improve decision-making quality by promoting more inclusive and comprehensive discussions. A multi-criteria decision analysis (MCDA) model was used to combine participants' varied opinions, data and judgments about the data's relevance to the issues at hand during a decision conferencing workshop where the priorities were finalised.

**Results:**

The study identified mental health interventions in three mental health priority areas: mental health inequalities, child and adolescent mental health, comorbidities with a focus on integration of mental and physical health services and mental health and substance misuse problems. Key interventions in all the priority areas are outlined. The programme is putting some of these evidence-based interventions into action nationwide in each of these three priority mental health priority areas.

**Conclusion:**

We report an inclusive attempt to ensure that the list of mental health service priorities agrees with perceived needs on the ground and focuses on evidence-based interventions. Other fields of healthcare may also benefit from this methodological approach if they need to make rapid health-prioritisation decisions.

## Introduction

There is a pressing need to address the burden of mental health and substance use disorders in England as mental health disorders, including anxiety, depression, and alcohol and substance use disorders, account for at least 21.3% of the burden of Years Lost to Disability in England (1). Although high-level priorities for future mental health research are regularly assessed (2, 3) and clinical practice guidelines for mental health problems are available, implementing evidence-based interventions into clinical practice remains challenging on multiple levels (4). Ineffective implementation leads to poor service delivery for people with mental health conditions and significant unmet needs (5), especially among deprived populations including ethnic minorities.

Despite recommendations to reform mental health systems (6, 7), progress towards adopting evidence-based interventions has been slow. To address this, the National Health Service (NHS) Mental health Implementation Plan was developed for England, setting out guidance for addressing inequalities and reducing this evidence-to-practice gap by 2023/24 (8). Additionally, new National Priority Programme, the Mental health Implementation Network (MHIN) (9), was established in 2020 under the National Institute for Health Research (NIHR) to understand the implementation and scale up of mental health interventions. As part of the MHIN programme, authors of this study were charged to identify mental health priority areas, identify specific interventions, and implement change in later phases to improve mental health service provision.

To reliably prioritise the unmet needs that evidence-based interventions can address, a rapid, systematic, and transparent process is required for our health systems (10). Traditionally, such processes involve expert panels who deliberate on a set of criteria and values deemed necessary for making transparent decisions (11–15). However, most priority-setting exercises in healthcare do not assess how practical it would be to implement prioritized interventions in the real world and often lack patients’, caregivers', and public's voices. Furthermore, with a few exceptions (including James Lind Alliance which is a non profit organisation that aims to bring patients, caregivers, and healthcare professionals together to identify and prioritize research topics in healthcare.), prioritisation exercises are rarely described in detail in the literature, making it difficult to learn and build better prioritisation processes. Therefore, a rapid, systematic, and transparent procedure on how to prioritize implementable interventions has become a recurring requirement for funders, policymakers, and implementers (5, 16).

The aim of this paper is to identify implementable evidence-based interventions in key mental health priority areas in England. The MHIN national prioritisation exercise was grounded on individual and broader system needs and evidence, and carried out with a focus on implementation. It is critical to identify mental health priority areas with the greatest unmet needs, which are supported by evidence-based interventions and are fit for delivery, especially in the current political climate in England, where improving mental health care access and “levelling up” are significant discourses.

## Methods

Our study aimed to address the complex prioritisation process of improving mental health services in England by utilising a mixed-methods research approach (17, 18).

The quantitative methods involved data collection through structured questionnaires and participant ratings. The data obtained through these methods were then analysed using descriptive statistics to summarise and interpret the numerical information. Additionally, we utilised MCDA to assess and weigh multiple criteria or factors involved in the decision-making process. On the other hand, the qualitative data collection focused on participant observations during decision conferencing sessions and narrative data gathered throughout the study. The qualitative analysis was guided by thematic analysis principles, which involved identifying recurring mental health priority areas, patterns, or concepts within the qualitative data.

By combining both qualitative and quantitative approaches, we sought to achieve a more comprehensive understanding of the phenomenon under investigation. To guide the study, an eight-member multidisciplinary steering committee consisting of MHIN investigators (PLJ, JP, LP, SA, CD, CS, JDM, TC), including experts by experience, was established.

**Figure 1:**
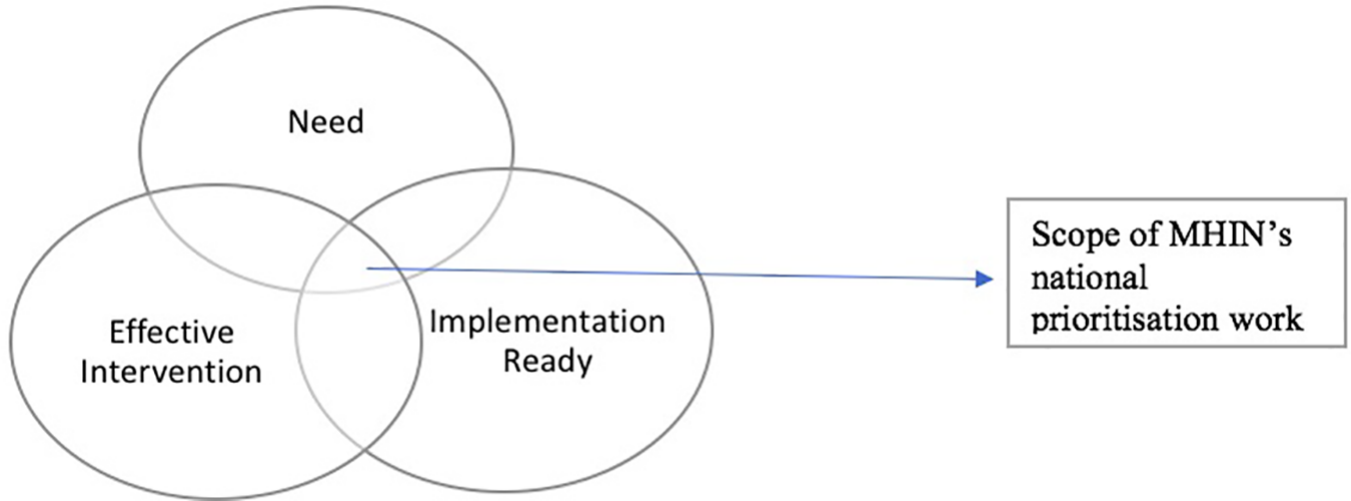
Scope of MHIN prioritisation work.

The steering committee determined the scope and process of prioritisation, which is illustrated in Figure 1. The scope of the work focused on identifying priority areas of unmet need, effective interventions, and ensuring implementation readiness. The “need” aspect of the scope refers to areas of mental health services that require improvement to meet the needs of individuals and the broader system. The “effective interventions” aspect of the scope refers to evidence-based solutions that have been proven to be effective in improving mental health outcomes. Lastly, the “implementation readiness” aspect ensures the proof of evidence of implementation of effective interventions through one or more healthcare providers in England.

**Figure 2:**
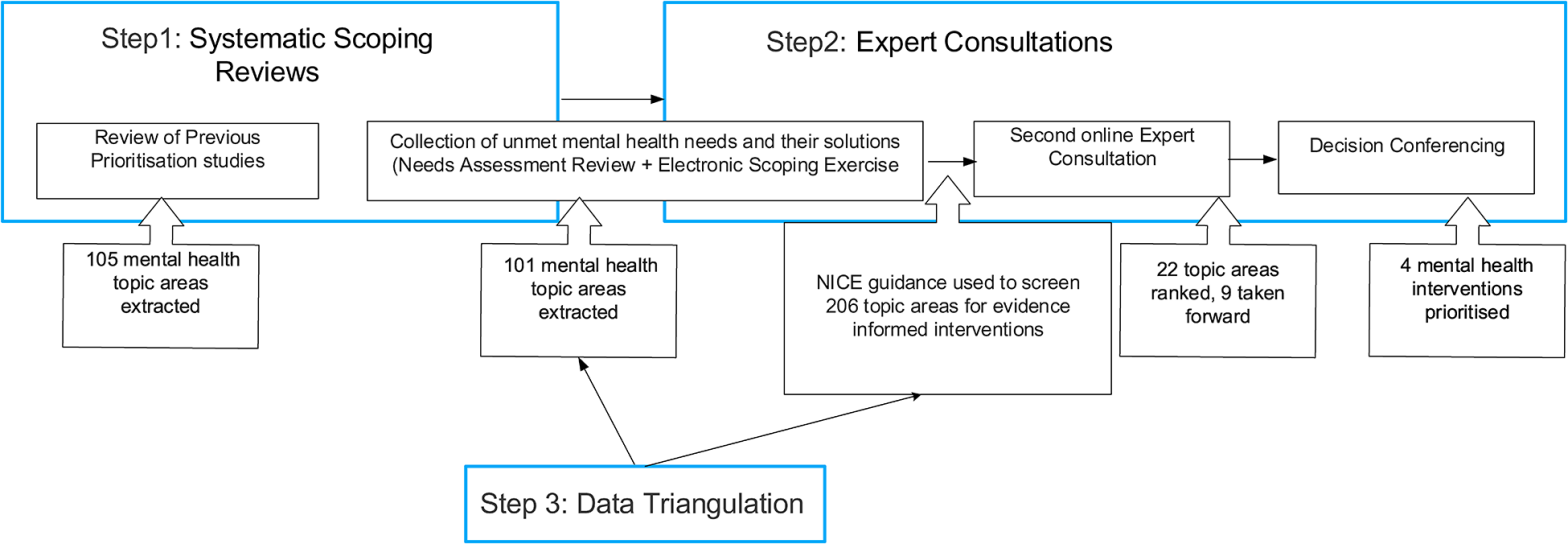
MHIN’S framework for prioritising mental health interventions for implementation.

To achieve our aims, the steering committee developed a comprehensive and three-step priority-setting process (as shown in Figure 2). This process involved a systematic scoping reviews, expert consultations, and data triangulation, which were iteratively refined. The systematic scoping reviews involved identifying and synthesising the best available evidence on unmet mental health needs in England. Expert consultations were conducted with a range of individuals, including mental health service users, caregivers, healthcare professionals, and policy-makers. Finally, data triangulation was employed to synthesise the findings of the systematic scoping reviews and expert consultations to identify priority areas for action. These three steps were not sequential as data triangulation was embedded within and across the first two steps of evidence search and consultations.

We conducted two systematic scoping reviews: a prioritisation review; and a needs-assessment review both of which followed the scoping-review methodology. A systematic approach was taken to the scoping review to identify knowledge gaps expand current understating of the concepts and inform next steps of this study (19). Prioritisation scoping review was conducted to identify topics identified as mental health research and service priorities in available reports and publications between 2015 and 2021, while a needs-assessment scoping review was conducted to identify mental health conditions with the highest burden of disease, unmet needs, variations in access, and health and well-being inequalities. The details of the two reviews are in a separate paper (manuscript in preparation).

The expert consultation process for mental health needs prioritisation was carried out in a rigorous and inclusive manner, spanning a period of six months and involving three distinct phases (see below). The process was used to comprehend diverse viewpoints and help build consensus among a group of stakeholders (20–22) and guided by the MHIN principles, which emphasised the importance of addressing mental health inequities, involving experts by experience and recognising mental health needs with evidence of implementation. The experts were consulted in three phases. All three expert consultations were conducted online, with both Phase 1 and Phase 2 being asynchronous, while decision conferencing took place live in a synchronous.

In the first phase of expert consultation process, an electronic scoping exercise was conducted to identify areas of perceived unmet needs in mental health service provision, with accompanying potential solutions. The steering committee used a structured survey to gather information from a wide range of relevant stakeholders across England, including mental health trusts, voluntary, social, and educational services, all 15 NIHR Applied Research Collaborations (ARCs), NIHR Translational Research Collaboration (TRCs), Academic Health Science Networks (AHSNs), NIHR Clinical Research Networks, and mental health-related charities and other third sector organisations. Perceived unmet mental health needs and service priorities were also identified via systematic scoping reviews (manuscript under process) which added to the list of priorities identified through this electronic scoping exercise. To reduce the number of priorities identified, the steering committee focused only on the largest mental health priority areas that aligned with national priorities and were endorsed by the National Institute for Health and Care Excellence (NICE), indicating that they were supported by evidence and could be considered for wider implementation.

In the next phase of expert consultation process, we organised second consultation exercise with similar stakeholder groups to further identify mental health priority areas. This step was introduced to sift the mental health priority areas to a manageable number. The stakeholders ranked mental health mental health priority areas according to five key objectives, including clinical effectiveness, involvement of patients and community in developing and delivering interventions, addressing health inequalities in terms of access to mental health services, implementation outcomes (23), and sustainability. The steering committee produced a list of evidence-based interventions with implementation evidence in each of the priority areas, referred to as implementable solutions. Furthermore, the Template for Intervention Description and Replication (TIDieR) (24) (Supplementary Appendix 5 for details on the checklist) checklist was used to characterise the specificities of implementable solutions in each topic area.

**Figure 3:**
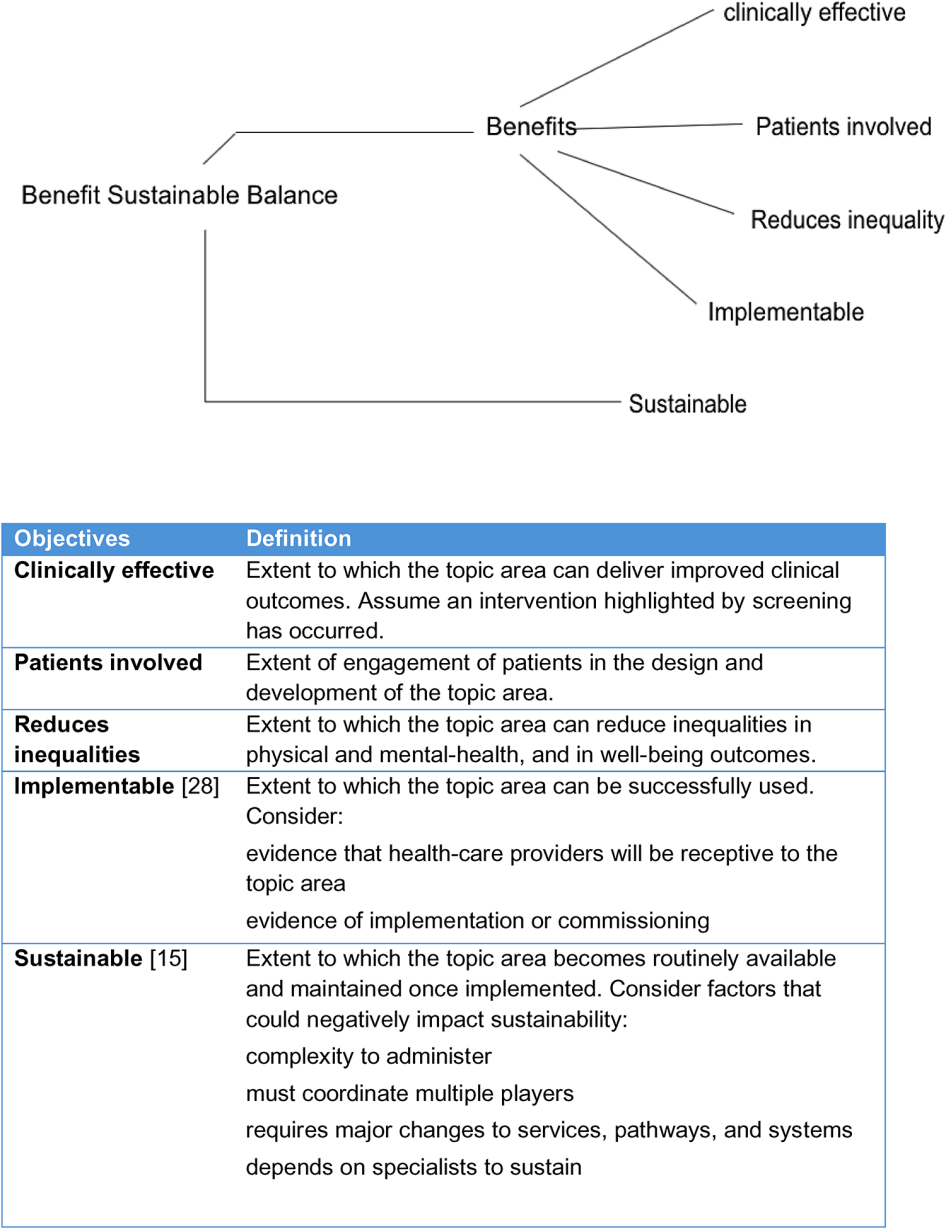
Value tree with objectives used to rank and score mental-health topic areas.

The third and final step of expert consultations, we engaged with 11 experts, plus the MHIN team, for a facilitated virtual workshop, known as a decision conference (25), to develop a multi-criteria decision analysis (MCDA) (26) model that established further established a final nine mental health priority areas. The approach disaggregates a complex problem into simpler problems, applies data and expert judgement, then uses mathematical logic to reassemble the pieces, giving guidance for the future work (27). It has been applied successfully to quantify the benefit-risk balance of drugs (28) and is increasingly used more generally in healthcare (26). The experts were selected for their diversity of experience with mental health issues and evaluated the mental health priority areas against each of the five objectives (see Figure 3 for objectives). The process ensured geographical representation, national/regional representation, third sector representation, specialty expertise (older adults, children and young people, experts from NHS-E/I, and expertise on inequalities and lived experience).

The social process of decision conferencing plus the technical modelling of MCDA enable a group of experts to move from implicit, qualitative perspectives about a problem to explicit, quantitative views in which preferences are constructed, debated, and agreed as new intuitions arise. They can lead to different, better differentiated, and more valid results than can be achieved by methods such as Delphi and Nominal Group Technique, as described in a comparison of the two approaches to research on the harm of drugs (29).

The most preferred topic area for a given criterion was assigned a value of 100, the least preferred topic area a value of zero, and the other seven mental health priority areas were assigned reference scores between 0 and 100, inclusive. The group also assessed criteria weights representing the differences in clinical value between least and most preferred mental health priority areas across the five objectives. The scoring for each objective proceeded in three phases: (1) identifying the most and least preferred mental health priority areas; (2) discussing a score for the next most preferred topic area; and (3) revealing and discussing the scores they were thinking of. This “think, reveal, discuss” process was intended to prevent participants from anchoring on the number suggested by the first person in an open discussion, and has been proven to minimise bias in group assessments (27). The five objectives are shown in the value tree of Figure 3, which was created using Hiview3 software (30). The “Sustainable” objective was separated because it could have conflicted with “Benefits” in the sense that while a topic might be rated as highly beneficial, it might also have low sustainability; this trade-off, represented by their normalised weights, could then easily be explored with the software. Scores and weights were entered into the Hiview3 software which normalised the weights so they summed to 100 over the five criteria, and then calculated weighted preference values that were summed across the five objectives to give an overall preference value for each topic area.

## Results

Below we present the findings from the three phases of expert consultations.

### Phase 1, electronic scoping exercise

In April 2021, 78 stakeholders completed the survey, out of a total of 190 stakeholders contacted. The stakeholder groups included ARCs, Biomedical Research Centres (BRCs), mental health trusts, TRCs, Local Clinical Research Networks (LCRNs), AHSNs, NICE, NHS E/I, and charities and other third sector organisations. The majority of respondents (47.5%) worked for mental health trusts, while the rest were employed by various organisations. A significant proportion (12.8%) of the stakeholders worked across these organisations or did not specify their organisation (see Table 1).

**Table 1:** Respondents involved in step 2 and phase 1 of the prioritisation process, which included expert consultations using the electronic scoping exercise.

Number of respondents	78 (41%)
Mental Health trusts (*n* = 54)	37 (47.44%)
Charities and other third sector organisation (*n* = 29)	9 (11.54%)
Local Clinical Research Networks (*n* = 15)	2 (2.56%)
Applied Research Collaborations (*n* = 15)	12 (15.38%)
Academic Health Science Network (*n* = 15)	2 (2.56%)
National Institute of Clinical Excellence	1 (1.28%)
Biomedical Research Centre (*n* = 20)	1 (1.28%)
NHS England/Improvement	3 (3.85%)
Others (*n* = 13)	10 (12.82%)

The respondents identified 92 perceived mental health needs in mental health priority areas. Among these needs, 60% of respondents recognised one unmet mental-health need, 25% identified two, 5% three, and approximately 2% identified four needs. These 92 perceived mental health needs, along with additional mental health needs from systematic desk reviews, were categorised into 17 different mental health priority areas or priority topics. These mental health priority areas included mental-health system strengthening, severe mental illness, comorbidities, trauma and crisis care, autism spectrum disorders and intellectual disabilities, learning disabilities, continuity of care (including early interventions), talking therapies and peer support therapies, self-harm and suicide, common mental disorders, technology-driven mental-health-care, eating disorders, inequalities, maternal mental-health, personality disorders, mental-health of older adults, and addictions.

**Figure 4:**
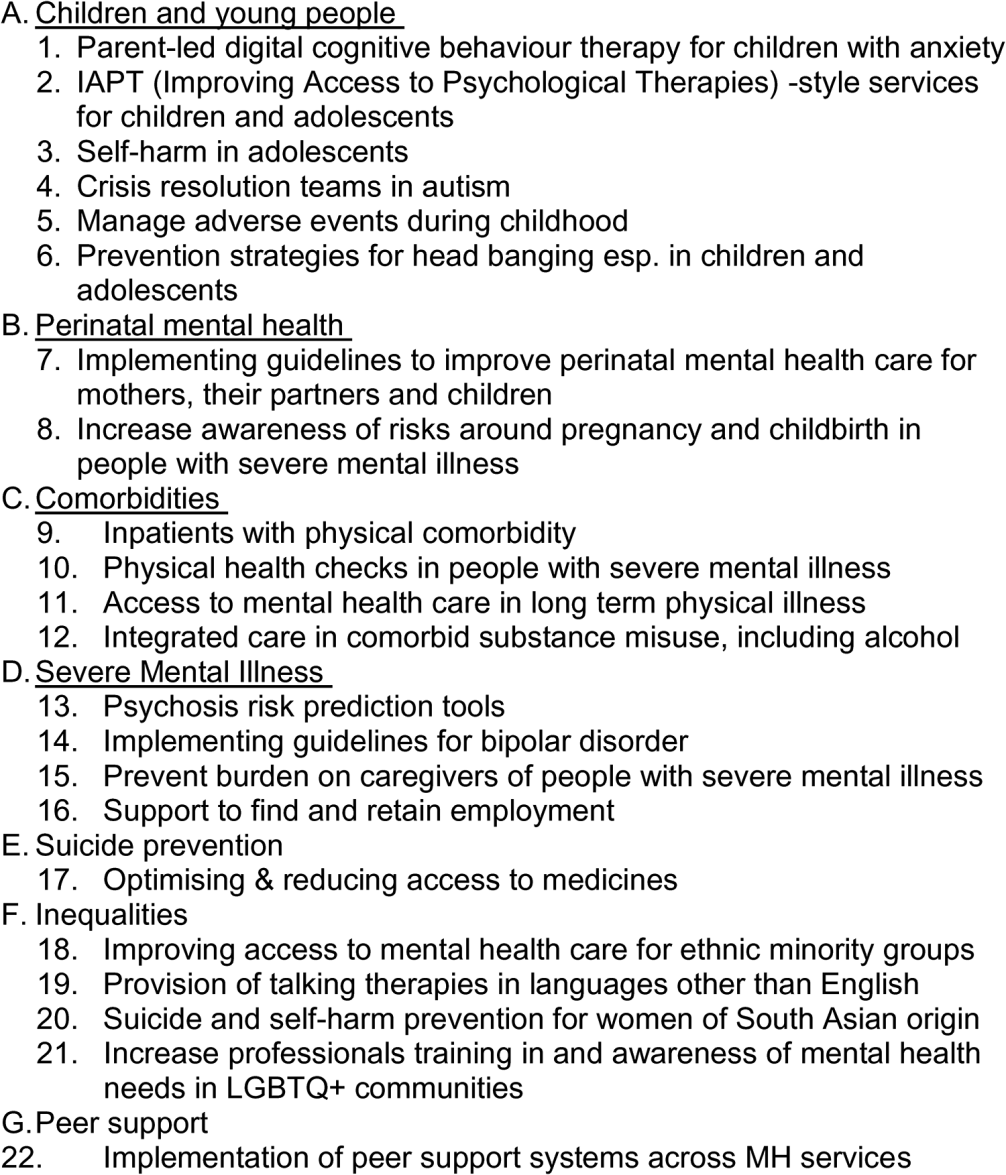
Top 22 mental-health needs.

Notably, all identified mental health needs where the proposed solutions did not align with NICE guidelines (31) and/or required structural modifications in the existing English mental health system were excluded. This exclusion at this stage suggested that the mental health priority areas may contain relevant interventions, but they lack the strength of evidence for broader implementation in the next phase of the MHIN programme. As a result, the number of unmet mental health needs was reduced to 22, which were grouped under seven key mental health priority areas. Figure 4 provides details of these 22 mental health priority areas.

### Phase 2, the second consultation exercise

The results from phase 2, the second consultation exercise conducted in May 2021 are presented in this section. A total of 196 stakeholders were contacted, of which 61 completed the survey. The response rate varied across different regions, with the East of England region recording the highest response rate. Mental health trusts (39.29%) were the most significant contributors among the different types of respondents, followed by universities (16.07%), ARCs, (10.71%), AHSNs (10.71%), charities (7.14%), TRCs (3.57%), NICE (1.79%), MHIN (1.79%), and ARC Well-being collaboration network (1.79%). The remaining organisations did not respond, and about 11% of the respondents identified themselves as “Other”. These results are presented in detail in Table 2.

**Table 2:** Response rate by ARC regions in step 2 and phase 2 of the prioritisation process, which included second online consultation with experts.

ARC region	Response rate 31% (*n* = 61/196)
East of England region	18.18%
Southwest Peninsula	10.91%
Oxford and Thames Valley	9.09%
Northeast and North Cumbria	7.27%
South London	7.27%
West Midlands	5.45%
Yorkshire & Humber	5.45%
East Midlands	5.45%
Kent, Surrey, and Sussex	3.64%
North Thames	3.64%
Northwest London	3.64%
Northwest Coast	3.64%
Wessex	3.64%
West	1.82%
Greater Manchester	No response
Others	10.9%

Mean priority scores for each of the five objectives, as well as an overall priority score, were calculated for each topic (score range, 1–5). Nine top-ranked topics were identified for the decision conferencing based on these scores, with mean scores ranging from 4.14 to 3.55 (see Table 3 for more details). These topics included physical health checks for people with severe mental illness, community engagement systems for people from racially minoritized community systems to improve their access to mental health services, mental health-care access for people with long-term conditions, Improving Access to Psychological Therapies (IAPT) style services for children and young people, employment support for people with severe mental illness, peer support systems across mental health services, suicide and self-harm prevention in South Asian women, management of patients with co-occurring severe mental illness and substance abuse, and psychosocial support for caregivers. Abbreviations were assigned to these topics for convenience, and these are presented in Table 4.

**Table 3:** Mean priority scores for mental-health topic areas (weighted average) in step 2 and phase 2 of the prioritisation process, which included second online consultation with experts.

Mental-health “Topic Area” type in rank order	Mean score
1: Physical health checks for people with severe mental illness, and consequent intervention.	4.14
2: Community engagement systems for people from racially minoritized communities to access mental-health-care.	4.13
3: Mental-health-care access for people with long-term physical conditions.	3.90
4: IAPT-style services for children and adolescents, especially at schools.	3.82
5: Support people with serious mental illness to find and retain employment.	3.78
6: Peer support systems across all mental-health services.	3.72
7: Suicide and self-harm prevention for South Asian women.	3.68
8: Integrated care protocols for patients with co-occurring serious mental illness and substance abuse.	3.62
9: Psycho-social support for caregivers of people with severe mental illness.	3.55

**Table 4:** Mental-health priority topic area type with short name used in step 2 and phase 3 of the prioritisation process, which included decision conferencing.

**PhysChks**: Physical health checks for people with severe mental illness, and consequent intervention. **MHC BAME**: Community engagement systems for people from racially minoritized communities to access mental-health-care. **MHCaccLT**: Mental-health-care access for people with long-term physical conditions. **IAPT Svcs**: IAPT-style services for children and adolescents, especially at schools. **Emplmnt**: Support people with serious mental illness to find and retain employment. **PeerSupp**: Peer support systems across all mental-health services. **HrmPrvSA**: Suicide and self-harm prevention for South Asian women. **InCrSubAb**: Integrated care protocols for patients with co-occurring serious mental illness and substance abuse. **CaregvrSup**: Psycho-social support for caregivers of people with severe mental illness.

For online stakeholders, the highest weighting amongst the five key objectives was given to clinical effectiveness (27%), followed by implementation (23%), reduction in health inequalities (20%), and engaging patients and the public (16%). The lowest overall weighting was given to the objective of capturing alignment with national priorities. A compendium of implementable interventions in each of the nine mental health priority areas was provided to the stakeholders for the decision conferencing to assist them in ranking the topics. These potential interventions are presented in detail in Supplementary Appendices 1–4.

### Phase 3, decision conferencing

It is worth noting that the MCDA process was a complex one. For example, one participant in the decision conference suggested, at the start of scoring, that all nine mental health priority areas were valuable, so the least and most preferred couldn't be established. However, the group soon agreed that the relative values differed from one objective to the next and they also realised that if their expertise was lacking for a particular topic area, they weren't required to contribute to the scoring. After the first hour of assessing preference values for the clinically effective objective, the group found that they could indeed assess quantitative preference values. MCDA modelling helped the group to collectively distinguish the greater value mental health priority areas from the lower value ones. The preference values assessed by the stakeholder group, where 100 identifies the most preferred topic area(s) and 0 the least preferred, one of each for every row, are detailed in Table 5. Note that 0 does not mean “no value”; it simply represents the option that is least preferred. The computer multiplied these scores by the normalised weights shown in the final column and summed the products in each column to obtain the total weighted preference value for each of the nine mental health priority areas. (Table 5 for more details).

**Table 5:** Preference values assessed by participants, criterion weights the right column, and the weighted preference values in the bottom row and the sum of the weights. (Step 2 and Phase 3 of the prioritisation process, which included decision conferencing).

	MHC BAME	PhysChks	IAPT svcs	MHCaccLT	CaregvrSup	InCrSubAb	Emplmnt	PeerSupp	HrmPrbsA	Weiwei
Clinically effective	70	100	100	90	50	70	30	0	20	21.5
Patients involved	100	0	80	20	90	70	65	100	10	16.7
Reduces inequalities	100	81	0	25	38	50	25	13	100	19.0
Implementable	100	50	70	80	70	0	20	70	30	23.8
Sustainable	70	100	70	80	0	20	30	0	20	19.0
Total	88	68	65	62	50	40	33	36	36	100

**Figure 5:**
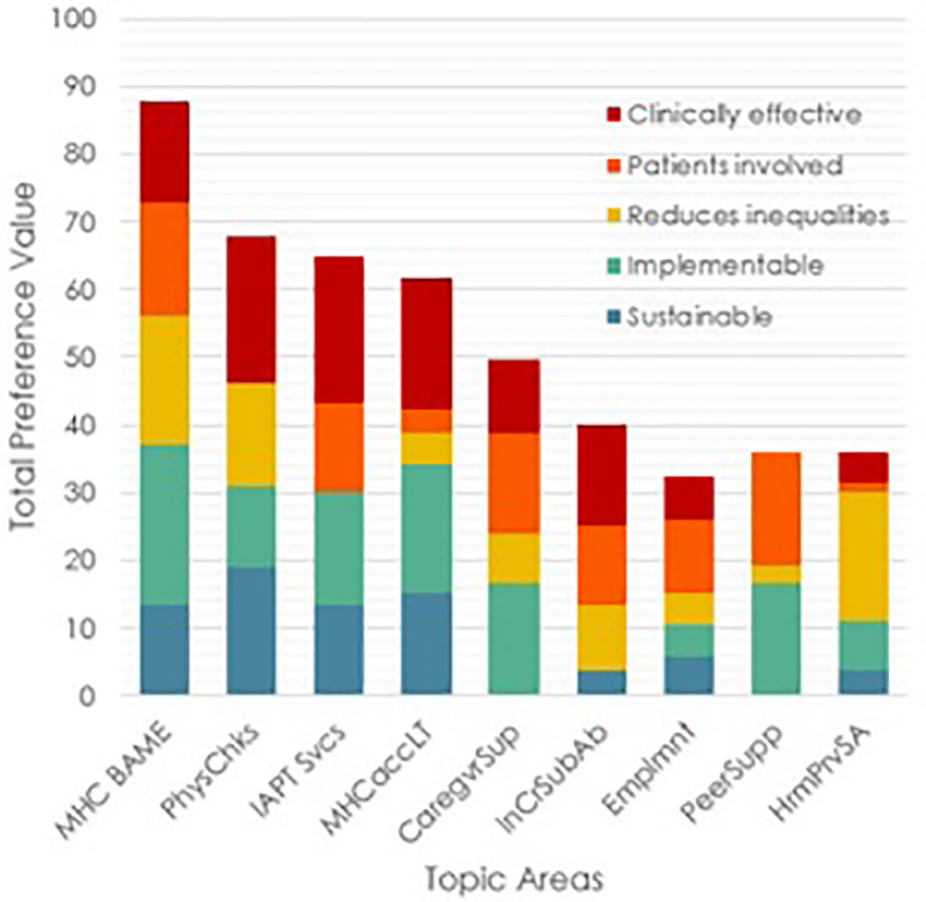
Bar graphs of the weighted preference values of the nine topic areas with the value contributions from each of the five criteria.

Figure 5 shows in graphical form those totals and their composition from the five objectives.

The MHIN network selected the three highest priority mental health priority areas and four sub themes for the next phase. The three priority areas are: mental health inequalities, child and adolescent mental health, comorbidities with a focus on integration of mental and physical health services and mental health and substance misuse problems. The sub theme selected in the mental health inequalities priority area is community engagement systems for people from ethnic minority groups to access mental health-care. The sub theme within the comorbidities priority area are two fold (a) physical health checks for people with severe mental illness and consequent intervention; integrated care protocols for patients with co-occurring serious mental illness and substance misuse. Finally, the sub theme within children and adolescents mental health priority area is IAPT-style services for children and adolescents. The MHIN steering committee decided to keep psycho-social support for caregivers of people with severe mental illness and mental-health-care access for people with long-term physical conditions as reserve mental health priority areas.

## Discussion

Inequity in health and social care, along with a higher level of mental health problems as a result of the COVID-19 pandemic, has resulted in an even greater of the mental health treatment gap (32, 33), which has been exacerbated by poor delivery of services within the NHS (34). Setting priorities for mental health services has become increasingly important, as revealed in an independent UK survey commissioned by the AHSNs (35). Our study put this into action by using a range of reproducible techniques for prioritising key mental health priority areas for the scale-up of mental health services in England.

### Mental health priority areas

Similar to other prioritisation exercises, three mental health priority areas such as mental health inequalities (21, 36), children and adolescent mental health (35, 37, 38), and comorbidities (including integration of mental and physical health (8, 38–40), and comorbidities (38) including mental health, and substance misuse (20, 41)) were identified in this study. Specifically, study proposes the following four sub themes in these three priority areas for implementation within the life of MHIN: (I) Community engagement systems for people from racially minoritized communities to access mental-health-care, (II) Physical health checks for people with severe mental illness, and consequent intervention, (III) IAPT-style services for children and adolescents, especially at schools, and (IV) Integrated care protocols for patients with co-occurring mental illness, long term physical illness and substance misuse.

While each of these mental health priority areas can be a collection of multiple interventions, many of them are secondary and tertiary level public mental health interventions since they incorporate evidence-based treatment for mental disorders. Although outside the scope of the MHIN, primary public mental health interventions and mental well-being promotion interventions across the life course should be equally explored (42).

### Theoretical considerations for priority-setting process

In several contexts, failure to scale up evidence-based mental health interventions is due to a lack of awareness of unmet needs, poor anticipation of implementation challenges, costs, and impact of interventions (42). Theoretical consideration of these principles at all stages, including prioritisation, can improve the odds of intervention acceptance and sustainment.

Therefore pivoting from traditional prioritisation approaches, this study made an inclusive attempt to incorporate some of these principles by (i) understanding perceived mental health needs on the ground by investigating the literature and incorporating stakeholders' views, (ii) focusing on evidence-based interventions which align with national priorities and provide information about implementation, and (iii) carefully selecting objectives for prioritisation that respond much better to implementation needs.

To nest this theoretical perspective, we used a mix of methodological approaches, including repeated expert consultations, thematic analysis and data triangulation which other health services research initiatives can learn from and emulate. The strong consensus reached in the various steps regarding mental health needs and the respective evidence-based solutions provides a solid foundation for other research initiatives in health and social settings in England, which in turn (and over time with appropriate resource) may improve mental health systems performance, at the national level.

Much of the prioritisation process convened various expert groups to elicit their judgments. However new research in the field of group decision-making (43) shows that involving diverse groups of specialists, including patient and public representatives and their dialectical inquiry increases decision-making quality. We believe that a comprehensive process such as ours which is supported by literature and engages with multiple stakeholders in decision-making using multiple objectives provided precision to our prioritisation process.

Overall, this exercise provides new and relevant information for health service researchers, policymakers, and implementation scientists on how to rapidly prioritise mental health areas with evidence-based interventions or service delivery models that have the potential to improve mental health services in the NHS. This effort of transparency and developing transferable methods adds to the novelty of this study. Our rapid prioritisation process took about 6 months and each priority area identified are further developed into interventions (see appendix for more details on interventions) and implementation strategies with greater involvement of experts by experience in the coming phases of the programme. All the sites will create implementation and evaluation plans and methods that are appropriate for the topic and local context, based on a variety of change methods and implementation theories, taking into account the complexity of the change required, as well as the scale of the ambition of implementation in terms of spread and sustainability.

### Study limitations

This study presents several limitations that must be acknowledged. First, this study, like other prioritisation exercises (44), highlighted both the necessity and the challenges of involving experts by experience (patient and public representatives) in priority setting exercises. The involvement of experts in decision-making is a critical aspect of ensuring transparency and accountability within the healthcare system (45). However, it is important to recognise that these representatives may not be representative of all service users and caregivers with unmet mental health needs. The authors of this study acknowledge this limitation and have taken steps to improve engagement with experts by experience throughout the prioritisation process and subsequently during implementation phase.

Secondly, respondents in the electronic scoping exercise and expert consensus survey were primarily asked to identify themselves with one organisation, which may have resulted in skewed response rates.

Thirdly, certain mental health mental health priority areas with high unmet needs and little or no evidence of implementation were excluded from the programme's remit, potentially resulting in the exclusion of emerging priorities or important issues that are difficult to measure. Future research could investigate these priority areas to address unmet needs.

Finally, the online nature of the prioritization process during the COVID-19 pandemic may have affected the response rate in expert consultations and potentially barred representation from digitally remote communities. For example, only 41% and 31%, respectively, of participants in the phase 2 and phase 3 of the expert consultations responded. It is important to consider all these limitations when interpreting the results of the study.

## Conclusions

This article reports on a prioritisation process designed to improve the evidence-based selection of mental health interventions. We have deployed a process for prioritising mental health interventions that embeds implementation thinking right from the beginning of the research cycle. The process includes assessing unmet needs, exploring implementation efforts of evidence-based solutions, engaging with key stakeholders, and providing specific recommendations to improve current prioritisation practices with a future goal of implementing these solutions within the life of the programme (3).

Although some may argue that each step in the prioritisation process could stand alone as a prioritisation exercise, the combination of approaches used has reduced the risk of bias and achieved the determination of an acceptable and defensible set of priorities. The process has also allowed us to reach out to a range of local and national stakeholders, and as a result, the need for improved communication and dissemination methods for patient groups and communities was identified. These established relationships will be critical in the subsequent steps of implementing the priorities.

We suggest that health-care organisations should recognise the relevance of research and appropriately allocate resources during the prioritisation of implementable health solutions. We also recommend that this approach can be used by others, not only in mental health, who need to make rapid and difficult health-prioritisation decisions with adequate resources.

## Data Availability

The original contributions presented in the study are included in the article/**Supplementary Material**, further inquiries can be directed to the corresponding author.
