# Supplementary material for: What interventions should we implement in England's mental health services? The mental health implementation network (MHIN) mixed-methods approach to rapid prioritisation
Source: Front Health Serv. 2023 Aug 11;3:1204207. doi: 10.3389/frhs.2023.1204207 (PMC10456870; doi:10.3389/frhs.2023.1204207)
Supplement: Supplementary file 1 [file Datasheet1.docx]

**Appendices**

**What interventions should we implement in England’s mental-health services? The Mental-Health Implementation Network (MHIN) mixed-methods approach to rapid prioritisation.**

Shalini Ahuja, Lawrence Phillips, Caroline Smartt, Sundus Khalid, Tina Coldham, Laura Fischer, Sarah Rae, Nick Sevdalis, Annette Boaz, Sarah Robinson, Fiona Gaughran, Zoe Lelliott, Peter Jones, Graham Thornicroft, Jayati-Das Munshi, Colin Drummond, Jesus Perez, Peter Littlejohns

**List of Appendicies**

Appendix 1: List of the interventions identified for the priority area: Implementation of community engagement systems to improve access to mental health care health for Black, Asian, and other minority ethnic groups.

Appendix 2: List of the interventions identified for the priority area: Integrated physical and mental health care for people with severe mental illness

Appendix 3: List of the interventions identified for the priority area: Improving Access to Psychological Therapies (IAPT)-style services for children and adolescents, especially in schools.

Appendix 4: List of the interventions identified for the priority area: Implementation of integrated care protocols for patients with co-occurring severe mental illness and substance abuse, including alcohol misuse

Appendix 5: TIDier (Template for Intervention Description and Replication) Checklist used as a template to document information of the evidence-based interventions identified.

Appendix 1: List of the interventions identified for the priority area: Implementation of community engagement systems to improve access to mental health care health for Black, Asian, and other minority ethnic groups.

| Implementation of community engagement systems to improve access to mental health care health for Black, Asian, and other minority ethnic groups | |
| --- | --- |
| Intervention | **Resource** |
| Patient and Carer Race Equality Framework (PCREF) | https://www.gmmh.nhs.uk/patient-carer-race-equality-framework-pcref-at-gmmh/ |
| Tackling Inequalities and Discrimination Experiences in health Services (TIDES) | https://tidesstudy.com/ |
| Culturally adapted Family Intervention for African Caribbean | http://research.bmh.manchester.ac.uk/ReACH/research/rfpb |
| Culturally adapted IAPT psychotherapies | <https://lewishamtalkingtherapies.nhs.uk/wp-content/uploads/2021/10/IAPT-BAME-PPG-2019.pdf> |
| Culturally adapted Cognitive behavioural therapy | http://legacy.synergicollaborativecentre.co.uk/wp-content/uploads/2020/12/The-importance-of-cultural-adaptation-IAPT-and-CBT.pdf |
| ROSHNI-2 | https://globalmentalhealthculturalpsychiatry.com/roshni-2/ |
| ARIADNE | https://mcpin.org/ariadne-addressing-the-impact-of-covid-19-pandemic-on-the-access-to-and-experience-of-mental-health-care-of-people-from-black-asian-and-minority-ethnic-groups/ |
| Ethnicity & Mental Health Improvement Project (EMHIP) | <https://emhip.co.uk/> |
| Healthy Minds Initiative | <https://www.nice.org.uk/sharedlearning/innovative-ways-of-engaging-with-black-and-minority-ethnic-bme-communities-to-improve-access-to-psychological-therapies> |
| Advance Decision-Making (ADM) | https://kclpure.kcl.ac.uk/portal/files/132294333/Stephenson_Gergel_et_al_2020_The_PACT_advance_decision_making_template.pdf |

Appendix 2: List of the interventions identified for the priority area: Integrated physical and mental health care for people with severe mental illness

| Integrated physical and mental health care for people with severe mental illness | |
| --- | --- |
| Intervention | **Resource** |
| National Clinical Audit of Psychosis (NCAP) Audit Core Standards | https://www.rcpsych.ac.uk/improving-care/ccqi/national-clinical-audits/national-clinical-audit-of-psychosis |
| PRIMROSE | https://www.ucl.ac.uk/psychiatry/primrose |
| Diabetes and SMI and self-management approaches | https://www.sciencedirect.com/science/article/pii/S2666560322000263 |
| Virtual Physical Health Clinic (VPHC) and Consultant Connect (CC) services | <https://implementationsciencecomms.biomedcentral.com/articles/10.1186/s43058-021-00113-0> |
| Collaborative Care (CoC) | https://www.england.nhs.uk/ourwork/patient-participation/patient-centred/c4cc/ |
| Implementing Recovery through Organisational Change (ImROC) | https://imroc.org/ |

Appendix 3: List of the interventions identified for the priority area: Improving Access to Psychological Therapies (IAPT)-style services for children and adolescents, especially in schools.

| Improving Access to Psychological Therapies (IAPT)-style services for children and adolescents, especially in schools | |
| --- | --- |
| Intervention | **Resource** |
| Brief Behavioural Activation in schools | https://www.cambridge.org/core/journals/behavioural-and-cognitive-psychotherapy/article/abs/brief-behavioural-activation-brief-ba-for-adolescent-depression-a-pilot-study/0D58F49E444B3B2FB8CFB2223FCDC2B1 |
| Parent-delivered CBT | https://www.researchgate.net/publication/256085143_Treatment_of_child_anxiety_disorders_via_guided_parent-delivered_cognitive-behavioural_therapy_Randomised_controlled_trial |
| i-THRIVE | https://implementingthrive.org/wp-content/uploads/2019/08/THRIVE-Framework-description-slides-FINAL.pdf |
| Culturally adapted IAPT psychotherapies | <https://lewishamtalkingtherapies.nhs.uk/wp-content/uploads/2021/10/IAPT-BAME-PPG-2019.pdf> |
| Brief Education Supported Treatment (BEST) | https://njl-admin.nihr.ac.uk/document/download/2030524 |
| Branching out | http://www.cambridgecandi.org.uk/projects/footprints/artscapers-being-and-becoming-creative#artscapers-being-and-becoming-creative |

Appendix 4: List of the interventions identified for the priority area: Implementation of integrated care protocols for patients with co-occurring severe mental illness and substance abuse, including alcohol misuse

| Implementation of integrated care protocols for patients with co-occurring severe mental illness and substance abuse, including alcohol misuse | |
| --- | --- |
| Intervention | **Resource** |
| Assertive outreach teams | https://nfao.org/Whats_New/Assertive_Outreach_Handbook.pdf |
| Realignment in commissioning structures | https://blogs.bmj.com/bmj/2017/11/30/return-failing-drug-and-alcohol-detoxification-services-to-nhs-control/ |

Appendix 5: TIDier (Template for Intervention Description and Replication) Checklist used as a template to document information of the evidence-based interventions identified.

| Item | Description |
| --- | --- |
| Brief Name | Provide the name or a phrase that describes the intervention |
| Why | Describe any rationale, theory, or goal of the elements essential to the intervention. |
| What | Materials: Describe any physical or informational materials used in the intervention, including those provided to participants or used in intervention delivery or in training of intervention providers. Provide information on where the materials can be accessed (e.g. Online appendix, URL).  Procedures: Describe each of the procedures, activities, and/or process used in the intervention, including any enabling or support activities. |
| Who provided | For each category of intervention provider (e.g. psychologist, nursing assistant), describe their expertise, background and any specific training given. |
| How | Describe the modes of delivery (e.g. face-to-face or by some other mechanism, such as internet or telephone) of the intervention and whether it was provided individually or in a group. |
| Where | Describe the type(s) of location(s) where the intervention occurred, including any necessary infrastructure or relevant features. |
| When and How much | Describe the number of times the intervention was delivered and over what period of time including the number of sessions, their schedule, and their duration, intensity or dose. |
| Tailoring | If the intervention was planned to be personalised, titrated or adapted, then describe the changes (what, why, when and how). |
| Modifications | If the intervention was modified during the course of the study, describe the changes (what, why, when and how). |
| How Well | Planned: If the intervention adherence or fidelity was assessed, describe how and by whom and by whom, and if any strategies were used to maintain or improve fidelity, describe them.  Actual: If the intervention adherence or fidelity was assessed, described the extent to which the intervention was delivered as planned. |
